# Genetic and epigenetic profiling of BRCA1/2 in ovarian tumors reveals additive diagnostic yield and evidence of a genomic BRCA1/2 DNA methylation signature

**DOI:** 10.1038/s10038-020-0780-4

**Published:** 2020-06-01

**Authors:** Erfan Aref-Eshghi, Jacob D. McGee, Victor P. Pedro, Jennifer Kerkhof, Alan Stuart, Peter J. Ainsworth, Hanxin Lin, Michael Volodarsky, Catherine Meg McLachlin, Bekim Sadikovic

**Affiliations:** 1 Molecular Diagnostics Division, Molecular Genetics Laboratory, London Health Sciences Centre, London, ON Canada; 2 Department of Obstetrics and Gynaecology, Western University, London, ON Canada; 3 Schulich School of Medicine and Dentistry, Western University, London, ON Canada; 4 Department of Pathology and Laboratory Medicine, Western University, London, ON Canada

**Keywords:** Diagnostic markers, Epigenomics

## Abstract

Poly-ADP-ribose-polymerase inhibitor (PARPi) treatment is indicated for advanced-stage ovarian tumors with *BRCA1/2* deficiency. The “BRCAness” status is thought to be attributed to a tumor phenotype associated with a specific epigenomic DNA methylation profile. Here, we examined the diagnostic impact of combined *BRCA1*/2 sequence, copy number, and promoter DNA methylation analysis, and evaluated whether genomic DNA methylation patterns can predict the BRCAness in ovarian tumors. DNA sequencing of 172 human tissue samples of advanced-stage ovarian adenocarcinoma identified 36 samples with a clinically significant tier 1/2 sequence variants (point mutations and in/dels) and 9 samples with a CNV causing a loss of function in *BRCA1*/*2*. DNA methylation analysis of the promoter of *BRCA1*/2 identified promoter hypermethylation of *BRCA1* in two mutation-negative samples. Computational modeling of genome-wide methylation markers, measured using Infinium EPIC arrays, resulted in a total accuracy of 0.75, sensitivity: 0.83, specificity: 0.64, positive predictive value: 0.76, negative predictive value: 0.74, and area under the receiver’s operating curve (AUC): 0.77, in classifying tumors harboring a *BRCA1/2* defect from the rest. These findings indicate that the assessment of CNV and promoter DNA methylation in *BRCA1*/*2* increases the cumulative diagnostic yield by 10%, compared with the 20% yield achieved by sequence variant analysis alone. Genomic DNA methylation data can partially predict BRCAness in ovarian tumors; however, further investigation in expanded *BRCA1/2* cohorts is needed, and the effect of other double strand DNA repair gene defects in these tumors warrants further investigations.

## Introduction

The application of chemotherapy as the standard treatment for advanced-stage ovarian cancer has been associated with little progress in improving the long-term clinical outcomes [1]. This has led to more targeted therapeutic approaches in the management of ovarian cancer including the use of poly-ADP-ribose-polymerase inhibitors (PARPis). PARP inhibitors induce targeted tumor cell death in homologous recombination repair-deficient cells (e.g., those with BRCA1/2 deficiency) through the exploitation of synthetic lethality [2]. Initial Phase-II clinical trials have shown a significantly longer progression-free survival in the PARPi-treated patients as compared with those receiving placebo (8.4 vs. 4.8 months), with a more pronounced effect in subjects that had germline or somatic *BRCA1/2* mutations (11.2 vs. 4.3 months) [3]. Subsequently, randomized controlled trials (RCTs) have shown improved progression-free survival in *BRCA1/2* mutation carriers when PARPis are used in the upfront maintenance setting (HR 0.3, 95% CI 0.23–0.41, P < 0.0001) and when used as maintenance in the recurrent setting (19.1 vs. 5.5 months, HR 0.30, 95% CI 0.22–0.41, *p* < 0.0001) [4, 5]. PARPis have thus been clinically approved for use in ovarian cancer patients with either germline or somatic *BRCA1/2* mutations [2, 4, 6].

Genetic testing is now being performed to detect somatic or germline mutations in *BRCA1*/2 to determine the eligibility for PARP inhibitors [6]. The detected variants are assessed according to the CAP/AMP classification guidelines [7], with tier 1 and tier 2 being eligible for PARPi therapy. For some patients, however, variants of uncertain clinical significance (VUS or Tier 3) are identified, which results in challenges in deciding on the appropriate therapeutic approach. In addition, some laboratories do not systematically evaluate other causes of BRCA deficiency such as copy-number variation (CNV) or aberrant promoter methylation of the *BRCA1*/2 genes. These events can induce an abnormal protein dosage comparable to the effect by sequence variants [8]. Therefore, a portion of the patients that benefit from PARPi therapy is not identified.

We have previously demonstrated that the implementation of copy-number variant assessment in clinical testing can significantly increase the detection yield in genetic testing [9]. We have also shown that haploinsufficiency of numerous genes in congenital disorders results in specific DNA methylation patterns across the genome, which can assist the diagnosis of the patients with an uncertain clinical diagnosis or those carrying VUSs [10–16]. DNA methylation profiling has frequently been used to classify tumor subtypes [17, 18] and prediction of treatment outcomes [19] in various cancers. In particular, a *BRCA1*-associated DNA methylation signature has been identified in the peripheral blood [20] and an attempt has been made to assess the pathogenicity of *BRCA1* unclassified genetic variants in breast cancer using DNA methylation profiling [21]. In ovarian cancer, it is established that tumors in which homologous recombination DNA repair defect is present, including those with BRCA’s deficiency, have a relatively distinct clinical and molecular phenotype, a concept referred to as BRCAness [22]. We hypothesize that such ovarian tumors have a distinct genomic DNA methylation pattern utilizing which, in conjunction with CNV and sequence variant assessment, can assist the identification of the patients who may benefit from PARP inhibitor therapy.

Here, we describe a comprehensive clinical testing approach for the assessment of the *BRCA1/2* genes in ovarian cancer specimens, which can be used to determine patients’ eligibility for PARP inhibitor therapy. We describe the overall clinical diagnostic yield as determined by sequence variant analysis and evaluate the improvements achieved by the incorporation of CNV and promoter DNA methylation evaluation of *BRCA1*/2. In addition, we describe a computational model for the assessment of *BRCA1/2*-associated genomic DNA methylation patterns in tumor tissues and examine the ability of this classifier to distinguish the patients that may benefit form PARP inhibitor therapy.

## Methods

### Patients and samples

Patient tissue specimens were obtained from tissue archive of deceased patients treated at the London Regional Cancer Program (LRCP, London, ON, Canada) between 2007 and 2012. All were under the age of 80 years, and treated for invasive high-grade serous, stage 3c or 4, epithelial ovarian carcinoma. Archival formalin-fixed paraffin-embedded (FFPE) slides of ovarian tissues from surgical resections were reviewed to confirm the diagnosis of high-grade serous carcinoma. Appropriate blocks with optimal tumor content were selected for analysis. This means that the cumulative tumor percentage of different blocks of each individual should be above 50%. Using a clean protocol (100% alcohol followed by DNAaway and then again with 100% alcohol), tissue blocks were cut at 20 μm sections with three sections, each placed in two tubes. Genomic DNA was then isolated using the Invitrogen RecoverAll total nucleic acid isolation kit (Thermo Fisher Scientific, Waltham, MA, USA) according to the manufacturer’s protocol. DNA quantification and genomic profile were then assessed with the 2200 TapeStation (Agilent Technologies, Santa Clara, CA, USA).

### High-throughput DNA sequencing

Extracted genomic DNA samples were placed into three groups based on genomic profile size ranges of <700 bp, 700–1100 bp, and 1100–2500 bp, and were subject to fragmentation to 180–220 bp using a Covaris E220 Series Focused-ultrasonicator (Covaris, Inc., Woburn, MA) with recommended settings and treatment times of 20, 50, and 60 s, respectively. Libraries were then prepared with the SeqCap EZ HyperCap workflow according to manufacturer’s protocol (Roche NimbleGen, Inc., Madison, WI, USA) and captured as a 24-plex pool with a custom target design that enriched for all coding exons, as well as 20 bp of the 5′ and 3′ flanking intronic regions for 37 hereditary cancer genes. Four captured libraries (96 samples) were diluted to 4 nM each and pooled for sequencing on the NextSeq according to the manufacturer’s protocol (Illumina, San Diego, CA, USA). The libraries were sequenced using the NextSeq version 2 mid output reagent kit to generate 2 × 150 bp paired-end reads. Post-sequencing file conversion generated FASTQ files for sequence alignment with the NextGene software version 2.4.1 (SoftGenetics, LLC, State College, PA, USA) using the recommended settings. The identified variants were filtered by an allelic fraction >10% and were assessed based on CAP guidelines for pathogenicity.

### Detection of copy-number variants

Base coverage distribution reports were created using NextGene software (SoftGenetics, LLC, USA) and processed through a normalization algorithm described previously [9] using a reference population of whole blood controls. Whole gene deletions and duplications for *BRCA1* or *BRCA2* that had at least a 30% allelic fraction were identified by concordance across four parameters: raw values (average normalized value per sample per gene), intra-sample ratio (ratio of average normalized value per gene of interest to average normalized value of the remaining genes on the panel (*n* = 36)), FFPE inter-sample ratio (ratio of average normalized value per gene to average normalized values of the same gene from other FFPE samples—Table S1), and whole blood inter-sample ratio (ratio of average normalized value per gene to average normalized values of the same gene from whole blood control samples). Sub-gene level events were identified by a minimum of 50% deviation from the normalized values of the remainder of the gene.

### Confirmation testing for sequence variants and CNVs

Sanger sequencing was performed on selected sequence variants (minimum 20% allelic fraction, PCR fragment size <300 bp based on current in-house primer stock) with the BigDye Terminator version 1.1 cycle sequencing kit (Life Technologies, USA). Sequencing products were separated by capillary electrophoresis on ABI 3730 (Life Technologies, USA) and were analyzed with Mutation Surveyor version 4.0.7 software (SoftGenetics, LLC, USA). Multiplex ligation-dependent probe amplification (MLPA) analysis was carried out for all copy-number variants >30% according to the manufacturer’s recommendations using SALSA MLPA kits P0002-BRCA1-D1 and P090-BRCA2-A4 (MRC Holland, Amsterdam, Netherlands). PCR products were separated by capillary electrophoresis on an ABI 3730 (Life Technologies) and analyzed with WB and FFPE references with Coffalyzer. Net software version 131211.1524 (MRC Holland).

### DNA methylation analysis

Of the samples processed in this study, 80 were selected for DNA methylation analysis. This included all of the samples with a clinically significant (Tier I/II) *BRCA1* or *BRCA2* variants together with a matching number of samples without any mutation in *BRCA1* or *BRCA2*. Following bisulfite conversion, DNA methylation analysis of the samples was performed using the Illumina Infinium methylation EPIC bead chip arrays, according to the manufacturer’s protocol. This array includes >860,000 human genomic methylation CpG sites, including 99% of RefSeq genes, all of the known disease-associated imprinted loci in humans, and 96% of CpG islands. The resulting methylated and unmethylated signal intensity data were imported into R 3.5.2 for analysis. Normalization was performed according to the Illumina normalization method with background correction using the minfi package. The methylation level for each probe was measured as a beta value, calculated from the ratio of the methylated signal intensity versus the total sum of unmethylated and methylated signal intensities for that probe, ranging between 0 (no methylation) and 1 (complete methylation). Probes with detection *p* values > 0.1, those located on chromosomes X and Y, those known to contain a SNP at the CpG interrogation or single nucleotide extension, and probes known to cross-react with chromosomal locations other than their target regions were excluded from analysis. All of the samples were examined for genome-wide methylation density to ensure evidence of bimodal distribution of the genomic DNA methylation levels. The majority of the samples were analyzed in one batch and only a few of them were processed in a different batch. Factor analysis using a principal component analysis was performed to examine the batch effect and identify potential outliers. No batch effect was observed between the samples processed among the two batches. Following quality controls, the methylation levels at the promoters of *BRCA1* and *BRCA2* were examined for an evidence of gain of methylation in the immediate 5′ promoter region in *BRCA1/2* mutation-negative specimens.

### Computational modeling of *BRCA1/2* positive samples

A fivefold cross validation using six different models and their ensembles was performed to examine whether the status of *BRCA1*/2 loss of function can be modeled using the DNA methylation data. The data were randomly divided into fivefolds. Fourfold was used for feature selection and model training while the remaining fold was used for testing the trained model. The process was repeated five times so that all of the folds were used for at least once during both testing and training. Feature selection was performed following three steps: (1) probes with a mean methylation difference of >0.05 between *BRCA1/2* positive and negative samples were retained; (2) probes were sorted based on the area under the receiver’s operating characteristic’s curve (AUC) and the top 1000 with the greatest AUC were retained; (3) a pair-wise correlation coefficient was measured for every probe, separately among the cases and controls, and highly correlated features (R-squared > 0.8) were excluded. Six different models were used for training including elastic net regression, support vector machine (SVM) with linear kernel, SVM with radial basis function kernel, linear discriminant analysis, random forest, and Bayesian generalized linear model. The ensemble of these models was conducted according to the pipeline implemented in the SuperLearner R package. Using a least absolute shrinkage and selection operator, each model was assigned a coefficient to be used for combining the predictions by multiple classifiers into the final classification. After repeating this procedure for all of the fivefolds, the average prediction accuracy measures on the testing set, including the total accuracy, sensitivity, specificity, positive predictive value (PPV), negative predictive value (NPV), and AUC were reported.

## Results

### Reportable sequence variants in *BRCA1* and *BRCA2*

DNA sequence analysis for *BRCA1* and *BRCA2* was conducted in a cohort containing 172 samples from patients with advanced-stage high-grade serous epithelial ovarian adenocarcinoma. The samples were analyzed using a custom-designed multi-gene NGS panel for *BRCA1* and *BRCA2*. The NGS analysis identified 36 samples with at least one clinically significant (Tier I/II) variant. Of these, 27 patients had a variant classified as Tier I (15 *BRCA1* mutations and 12 *BRCA2* mutations), with 1/27 having both a Tier I and a Tier II variant and 5/27 having both a Tier 1 and Tier III variant. There were also six samples with only VUS (Tier III) in *BRCA1* or *BRCA2* (Table 1).

**Table 1:** Reportable sequence variants and CNVs in *BRCA1* and *BRCA2*

Sample ID	Tier 1	Tier 2	Tier 3 (VUS)	CNVs
1105-001*	BRCA1:c.3254_3255dupGA, p.(Leu1086Aspfs*2) (57.6%)			
1105-009*	BRCA1:c.1961dupA, p.(Tyr655Valfs*18) (33.9%)			
1105-039*				BRCA1 exon13dup
1105-054*	BRCA1:c.5266dupC, p.(Gln1756Profs*74) (58.4%)			
1105-057*	BRCA1:c.5095C>T, p.(Arg1699Trp) (44.2%)			
1105-112*	BRCA1:c.5207T>C, p.(Val1736Ala) (84.7%)			
1105-155*	BRCA1:c.4327C>T, p.(Arg1443*) (46.1%)			
1105-162*	BRCA1:c.212+3A>G (96.7%)			
1105-195*	BRCA1:c.5059delG, p.(Val1687Leufs*3) (51.3%)			
1105-207*				BRCA1del
1105-227*	BRCA1:c.5266dupC, p.(Gln1756Profs*74) (40.6%)			
1105-270*		BRCA1:c.2891delG, p.(Gly964Aspfs*36) (58.4%)		
1105-273*	BRCA1:c.4621G>T, p.(Glu1541*) (31.7%)			
1105-024*	BRCA2:c.7615C>T, p.(Gln2539*) (11.1%)	BRCA1:c.2423_2481del, p.(Phe808Trpfs*3) (27.1%)		
1105-204*				BRCA1del
1105-110*	BRCA1:c.1116G>A, p.(Trp372*) (80.1%)			
1105-113*		BRCA1:c.182G>C, p.(Cys61Ser) (89.2%)		
1105-119*	BRCA1:c.3967C>T, p.(Gln1323*) (93.3%)		BRCA2:c.9665G>T, p.(Cys3222Phe) (24%)	
1105-123*	BRCA1:c.5266dupC, p.(Gln1756Profs*74) (69.3%)			
1105-130*				BRCA1del
1105-159*		BRCA1:c.5096G>A, p.(Arg1699Gln) (44.6%)		BRCA2dup
1105-185*				BRCA1 exon13dup
1105-192*	BRCA1:c.68_69delAG, p.(Glu23Valfs*17) (76.3%)			BRCA2dup
1105-193*		BRCA1:c.3394_3406del, p.(Asn1132Leufs*19) (70%)		BRCA1dup
1105-222*		BRCA1:c.709G>T, p.(Glu237*) (51.2%)		
1105-246*		BRCA1:c.5074G>A, p.(Asp1692Asn) (72.8%)		
1105-049*	BRCA2:c.7958T>C, p.(Leu2653Pro) (99.4%)			
1105-088*	BRCA2:c.3170_3174del, p.(Lys1057Thrfs*8) (71.4%)			
1105-103*	BRCA2:c.6591_6592delTG, p.(Glu2198Asnfs*4) (53.7%)		BRCA2:c.9305C>T, p.(Ala3102Val) (43.1%)	
1105-104*	BRCA2:c.7954delG, p.(Val2652Cysfs*5) (38.5%)			
1105-111*	BRCA2:c.8167G>C, p.(Asp2723His) (69.6%)		BRCA1:c.-62G>A (57.1%)	
1105-128*				BRCA2del
1105-152#	BRCA2:c.3545_3546delTT, p.(Phe1182*) (34%)			
1105-200*	BRCA2:c.7617+2T>G (63.7%)			
1105-202*	BRCA2:c.2588dupA, p.(Asn863Lysfs*18) (67.2%)		BRCA1:c.-86C>T (83.3%)	
1105-265#*	BRCA2:c.633dupC, p.(Arg212Glnfs*3) (45.2%)			
1105-139*		BRCA2:c.3587T>A, p.(Leu1196*) (73.3%)		
1105-078*		BRCA2:c.8164dupA, p.(Thr2722Asnfs*8) (37.3%)		
1105-137*	BRCA2:c.7069_7070delCT, p.(Leu2357Valfs*2) (18.7%)		BRCA2:c.1094C>T, p.(Pro365Leu) (17.6%)	
1105-144*				BRCA2del
1105-258*	BRCA2:c.1054dupT, p.(Tyr352Leufs*6) (72.2%)			
1105-268*				BRCA2del
1105-041	BRCA1:c.4258C>T, p.(Gln1420*) (11.9%)			
1105-182		BRCA1:c.5497G>A, p.(Val1833Met) (10.3%)	BRCA2:c.10112C>T, p.(Thr3371Ile) (10.5%)	
1105-036				BRCA1dup
1105-018				BRCA1dup
1105-022			BRCA2:c.9338T>C, p.(Ile3113Thr) (75.6%)	
1105-055				BRCA2dup
1105-059			BRCA1:c.4531C>T, p.(His1511Tyr) (12.2%)	
1105-120			BRCA2:c.44_45insATT, p.(Ile14_Phe15insLeu) (45.7%) BRCA2:c.9502–12T>G (82.5%) BRCA2:c.41_67+9del, p.(?) (12.4%)	
1105-126			BRCA2:c.2716A>G, p.(Thr906Ala) (66.1%)	
1105-157				BRCA2dup
1105-174			BRCA1:c.2212G>A, p.(Val738Ile) (10.2%)	
1105-178			BRCA1:c.5416C>T, p.(Pro1806Ser) (14.7%)	
1105-181				BRCA2dup
1105-183			BRCA1:c.2713C>G, p.(Gln905Glu) (23.9%)	BRCA2dup
1105-197				BRCA1dup
1105-198				BRCA2dup
1105-220			BRCA2:c.5714A>T, p.(His1905Leu) (43.1%)	
1105-229				BRCA2dup
1105-277			BRCA2:c.9698G>C, p.(Cys3233Ser) (47.7%)	

Percentages in brackets indicate alternate allele fractions. Due to the presence of varying levels of normal tissue in these tumor specimens, as well as confounding factors such as chromosomal aneuploidy or copy-number alterations, the true allele fractions in the tumor may be above what is indicated. Samples with allele fractions above 40% were considered for PARPi therapy. Most of the samples had a tumor percentage >50%. It is notable that this number may not indicate a heterozygous state in the heterogenous tumors. The samples with residual or low tumor volume (10–20%) are indicated with a hash sign. The copy-number changes were confirmed with MLPA. Gene duplications are not assessed clinically, since they are not confirmed to result in recombination deficiency. Samples with a Tier 1 or Tier 2 variants or those with truncating intragenic duplications/full gene deletions are eligible for PARPi therapy. Samples used in methylation testing are indicated with an asterisk

### *BRCA1* and *BRCA*2 copy-number variants

The CNV analysis identified a total of ten subjects with a CNV involving *BRCA1* (four full gene duplications, four full gene deletions, and two *BRCA1* exon 13 duplications—Table 1). In addition, there were 11 cases with a CNV overlapping *BRCA2*, including 8 samples with duplications and 3 with deletions of the entire gene. Among none of the samples with deletions (duplications are not eligible for PARPi therapy), the primary sequence variant assessment had not identified a reportable sequence variant (Table 1).

### DNA methylation analysis of the *BRCA1/2* promoters

**Fig. 1:**
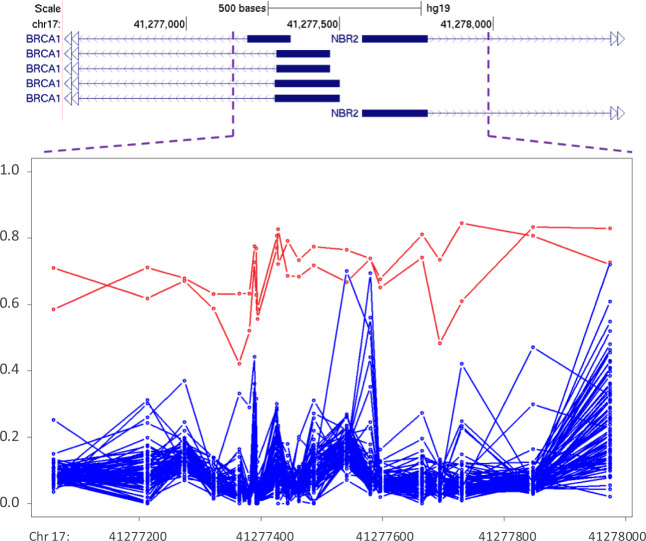
DNA methylation analysis of *BRCA1* promoter. The figure illustrates >500 bps annotating to the promoter of the *BRCA1* gene in individuals with no sequence variant findings in *BRCA1*/2. The methylation levels for each CpG site in this region (0–1) is shown using a circle, connected to the adjacent CpGs of the same individual using a line. The majority of samples show a hypomethylation pattern (blue), while two samples show a gain of methylation (red)

A total of 130 samples were deemed negative for any *BRCA1* and *BRCA2* reportable sequence variants or CNVs, among which 38 were subject to DNA methylation testing. The methylation analysis of the promoters of *BRCA1* and *BRCA2* identified two additional patients with increased methylation levels of up to 60% (consistent with hypermethylation of one of the two alleles) as compared with the rest of the samples, all of which were fully hypomethylated (median cross-region methylation level <15%, Fig. 1). This indicated a 5% increase in the diagnostic yield of samples negative in *BRCA* sequence variant testing. In addition, none of the patients with a reportable sequence variant in BRCA’s showed hypermethylation in the promoter of *BRCA1* or *BRCA2*, indicating that the patients with BRCA’s loss of function due to pathogenic variants, do not have additional promoter hypermethylation.

### Classification of ovarian tumors by BRCA status using DNA methylation data

We attempted, using an ensemble learning approach, to determine whether samples with and without *BRCA1*/2 mutations can be classified using the genome-wide DNA methylation data. DNA methylation profiling was performed on a total of 44 samples with *BRCA1*/2 loss of function variants and 36 samples without a reportable variant or a promoter DNA methylation aberration in *BRCA1*/2. Following feature selection, six different classification models were developed among which SVM with linear kernel reached the highest accuracy and was most consistently selected to be included in the final ensemble. Therefore, at the ensemble, the predictions of most models other than SVM were down-weighted. The average classification accuracies of the final model as determined during the fivefold cross-validation on the testing set—not used for feature selection or model training—were as follows: total accuracy: 0.75, sensitivity: 0.83, specificity: 0.64, PPV: 0.76, NPV: 0.74, and AUC: 0.77 (Tables 2, S2, and S3). These results indicated that DNA methylation data can partially predict the status of BRCA deficiency in ovarian cancer tumors.

**Table 2:** Accuracy measures for classification of ovarian tumors by BRCA status using DNA methylation data

Accuracy indicator	Fold 1	Fold 2	Fold 3	Fold 4	Fold 5	Average
Overall accuracy	0.69	0.81	0.81	0.71	0.73	0.75
Sensitivity	0.67	0.89	0.89	0.8	0.89	0.83
Specificity	0.71	0.71	0.71	0.57	0.5	0.64
PPV	0.75	0.8	0.8	0.73	0.73	0.76
NPV	0.62	0.83	0.83	0.67	0.75	0.74
AUC	0.68	0.79	0.79	0.8	0.78	0.77

## Discussion

Utilization of high-throughput sequencing has become a common practice in the clinical testing of constitutional and somatic disorders. These assays enable for thorough screening of single nucleotide variants and short indels, while larger indels, extensive copy-number changes, and other causes of gene function aberrations, such as defects in DNA methylation, remain unexplored in most clinical assessments. An example of this applies to the genetic testing of *BRCA1*/2 in ovarian cancer. In this study, we have demonstrated that ~20% of the ovarian tumors can be identified with a pathogenic SNV/indel in *BRCA1*/2 while another ~10% will have a pathogenic CNV or a promoter DNA methylation defect in these genes. DNA methylation testing, despite the documented effect on PARPis response rates [23], is not being conducted in the routine assessment of drug eligibility. Thus, a multi-faceted approach can have a greater potential in detecting patients that may benefit from PARPi therapy.

Currently, there is no established guideline regarding the use of PARPis in ovarian tumors with BRCA promoter hypermethylation. However, new evidence suggests efficacy of this class of drugs on tumors with such aberrations. Swisher et al. have reported up to 50% response rate for Rucaparib, a PARP inhibitor, in ovarian samples that had *BRCA1* promoter hypermethylation [23]. In their cohort of 165 ovarian samples, 12% had hypermethylation in the *BRCA1* promoter, a figure comparable to those with sequence variant defects. Another study has found that none of the ovarian tumors with *BRCA1* promoter hypermethylation demonstrate BRCA1 protein expression by immunohistochemistry, being consistent with the silencing of the *BRCA1* gene [8]. This indicates a possible new target patient population that could benefit from PARPis. The effectiveness of PARPis in samples with *BRCA1/2* promoter hypermethylation has also been documented in other tumors, including a clinical trial currently undergoing for triple-negative breast tumors with *BRCA1/2* hypermethylation [24].

Another way DNA methylation testing can benefit the identification of patients eligible for PARPi’s is through the use of a DNA methylation profile associated with the BRCAness phenotype. Ovarian tumors with BRCAs mutations are known to have a distinct clinical behavior, mainly attributed to homologous recombination deficiency, leading to massive genomic instability and affecting many layers of the regulation of gene expression such as DNA methylation [25]. A DNA methylation pattern specific to BRCAness can be used to resolve uncertain cases where, for instance, a VUS is found in *BRCA1*/2. Our analysis shows that the status of *BRCA1*/2 mutations can be, to some extent, modeled using genomic DNA methylation data. The heterogeneous nature of ovarian tumors, however, may not enable a full accuracy in the classification of the samples into *BRCA1*/2 positive/negative profile. A previous study using DNA methylation data has also reached a comparable accuracy (~80%) in detecting breast tumors with *BRCA1* mutations [26]. A BRCAness phenotype can be present in tumors with homologous recombination deficiency, but without a defect in BRCA’s expression [27]. It is possible that some of the non-mutated BRCA tumors in these studies have had a BRCAness profile, limiting the accuracy of DNA methylation classification. Consistently, the utility of PARPis is shown to be not limited to BRCA1 and BRCA2 deficient tumors. Any defective homologous recombination caused by the deficiency of other genes can be a potential target for PARPis therapy. Proteins involved in homologous recombination repair other than BRCA1 and BRCA2, including ATM, CHEK2, BARD1, BRIP1, MRE11, RAD50, NBS1, RAD51C, RAD51D, and PALB2, have been reported to be associated with increased susceptibility to ovarian cancer. Several of these have now been associated with moderate increased susceptibility to ovarian and/or breast cancer [22, 23, 27–32]. Swisher et al. have reported a 75% response rate by PARPis for RAD51 deficient ovarian tumors [23]. On a non-gynecological cancer example, there is emerging evidence that the use of PARPis can potentiate the action of alkylating agents in glioblastoma tumors that have hypermethylation of the promoter of *MGMT*, coding for another DNA repair protein [33].

These findings all raise the question of whether a DNA methylation signature of homologous recombination deficiency could be used to better identify patients in need of PARPi therapy than sequence variant assessment of *BRCA1* and *BRCA2*. Further analyses on larger sample sizes attempting to map a DNA repair-associated DNA methylation signature that includes all of the involved genes beyond BRCAs, such as *ATM, CHEK2, BARD1, BRIP1, MRE11, RAD50, NBS1, RAD51C, RAD51D, and PALB2*, followed by clinical trials to evaluate the effectiveness of PARPis in the group of patients showing such a methylation profile are needed before the implementation of DNA methylation profiling in clinical testing of ovarian cancer patients.

A final point to consider about the PARPi eligibility is about the somatic vs. germline and zygocity status of the genetic testing findings. While it is expected that a proportion of PARP-sensitive tumors exhibit biallelic loss of BRCA1/2 that may be detectible by this analysis, in the majority of cases, the identified mutation, deletion, or methylation defects appear to occur in a single allele. Based on the recommendations by the Pan-Canadian Oncology Drug Review expert review committee, Olaparib monotherapy maintenance treatment is recommended for BRCA1/2-mutated patients with an evidence of a germline or somatic defect as detected by approved testing laboratories [34]. Therefore, while this assay is not designed to determine the germline vs. somatic status or biallelic loss of BRCA, it meets the requirements for the identification of PARP-eligible individuals.

## Supplementary information

Supplementary Material
